# Parametric Analysis to Study the Influence of Aerogel-Based Renders’ Components on Thermal and Mechanical Performance

**DOI:** 10.3390/ma9050336

**Published:** 2016-05-04

**Authors:** Sofia Ximenes, Ana Silva, António Soares, Inês Flores-Colen, Jorge de Brito

**Affiliations:** Instituto Superior Técnico, University of Lisbon, Lisbon 1049-001, Portugal; sofia.ximenes@ist.utl.pt (S.X.); anasilva931@msn.com (A.S.); ortiz.soares@gmail.com (A.S.); jb@civil.ist.utl.pt (J.d.B.)

**Keywords:** renders, aerogel, performance, experimental study, parametric analysis, SPSS

## Abstract

Statistical models using multiple linear regression are some of the most widely used methods to study the influence of independent variables in a given phenomenon. This study’s objective is to understand the influence of the various components of aerogel-based renders on their thermal and mechanical performance, namely cement (three types), fly ash, aerial lime, silica sand, expanded clay, type of aerogel, expanded cork granules, expanded perlite, air entrainers, resins (two types), and rheological agent. The statistical analysis was performed using SPSS (Statistical Package for Social Sciences), based on 85 mortar mixes produced in the laboratory and on their values of thermal conductivity and compressive strength obtained using tests in small-scale samples. The results showed that aerial lime assumes the main role in improving the thermal conductivity of the mortars. Aerogel type, fly ash, expanded perlite and air entrainers are also relevant components for a good thermal conductivity. Expanded clay can improve the mechanical behavior and aerogel has the opposite effect.

## 1. Introduction

In order to reduce buildings’ energy consumption, new solutions have been developed to improve their thermal performance. The development of thermal mortars is one of the available solutions that contribute to energy efficiency in construction [1,2].

In addition to reducing energy costs in heating and cooling, thermal mortars may also minimize production costs by having in their composition organic, natural, or recycled materials [3,4,5,6,7,8,9].

The addition of lightweight aggregates and binders, which lower a render’s density, is an option to improve the thermal behavior of renders [10].

Aerogels arise in this context as very lightweight materials with excellent thermal properties [11]. The knowledge of silica aerogel incorporation in mortars has grown, but there are still a lot of questions concerning the content and render components required to obtain high thermal performance with acceptable mechanical performance.

In this study, 85 mortar mixes with aerogel incorporation were subjected to several laboratory tests within a research project at IST (Instituto Superior Técnico). The components’ content varied in order to obtain mixes with thermal mortar characteristics, affecting as little as possible the typical mechanical properties of a render. Later, the influence of various components on the thermal and mechanical performance of mortars with aerogel incorporation was analyzed. Regarding the high amount of different renders necessary to accomplish this analysis, a different approach was used to understand the influence of each component. For that purpose, a multiple linear regression technique was used, which is not usually applied in other typical studies in this area. Using this tool, a statistical model of the thermal and mechanical performance (using the experimental values of the thermal conductivity coefficient and compressive strength, respectively) was developed, thereby allowing statistically analyzing the influence of the components and their importance on the performance of aerogel-based renders.

## 2. Literature Review

The energy and environmental European Directives (Directives 2002/91/EC [12] of 16 December 2002, and 2010/31/EU of 19 May 2010 [13]) have contributed to the development of alternatives in the construction industry and, consequently, to the pursuit of sustainability. With increasing demand for comfort, there has been a high energy consumption associated with the air conditioning of housing units. Thermal renders contribute to reducing these energy costs [1,2]. They have a low thermal conductivity coefficient, but are able to maintain their coating functions. According to standard EN 998-1 [14], thermal renders’ maximum thermal conductivity coefficient is 0.1 W/m·K (Class T1) and 0.2 W/m·K (Class T2).

In this context the number of studies on new solutions that intend to minimize the incorporation of processed raw materials and energy consumption, as well as the resulting environmental impact of the production, use, and disposal of the product at the end of its service life, has been increasing [1]. To improve the performance of renders, sustainable and innovative lightweight materials and/or materials with thermal insulation properties, such as recycled aggregates and/or nano-structured materials, are incorporated [3,4,5,6,7,8,9,15,16,17,18,19].

The incorporation of lightweight aggregates improves the renders’ thermal performance and significantly reduces their bulk density in the hardened state, allowing them to be classified as lightweight renders according to EN 998-1 [14] with a density lower than 1300 kg/m^3^.

The replacement of sand with expanded cork granules improves the thermal characteristics of renders due to their low thermal conductivity coefficient, between around 0.042 and 0.070 W/m·K [19,20]. Cork is also a very low density material (between 100 and 140 kg/m^3^) and is expected to contribute to achieving lightweight renders [19]. Expanded clay is used as a building material because of its high physical and chemical stability and low cost [19,21]. It has a low density (between 300 and 700 kg/m^3^), a high porosity, and a thermal conductivity of approximately 0.10 W/m·K [19,22].

It is also possible to obtain better thermal performance by changing the binders, bearing in mind that the thermal conductivity decreases as the density goes down [10]. The incorporation of fly ash is also beneficial to reduce the render’s thermal conductivity [10] but causes a reduction on mechanical strength [23]. Due to its fineness, lime has important plasticizing and water retention properties. In addition to these characteristics, a mixed cement plus lime binder can increase the render’s porosity, leading to lightweight renders [24]. Lime allows lowering the thermal conductivity, while cement improves the mechanical properties [24,25].

The incorporation of air entrainers, resins, and rheological agents, at adequate ratios, can also improve the performance of renders. The air entrainers allow increasing porosity, and the resins and rheological agents improve the bond of the components and may also contribute to a decrease of the thermal conductivity [26,27].

Access to nanotechnology, regarding the handling of materials with size and precision between 0.1 and 100 nm [28], enabled improving the physical and chemical properties of conventional products by changing their microstructure [29,30]. Nanomaterials are being used in mortars to improve given properties [15,31]. This is the case of carbon nanotubes that prevent cracking [16,32], silica nanoparticles that contribute to increase strength [18,28], emulsions that improve the water absorption and graffiti paint resistance [33], and titanium dioxide that provides aesthetic protection to the surface level [15,34,35]. Aerogels are high-porosity mineral materials, mainly composed of air that can be produced in powder or granular form, leading to significant improvements of the products where they are incorporated [11]. Aerogels are extremely lightweight materials (density less than 500 kg/m^3^), with excellent thermal properties (a thermal conductivity that can reach 0.01 W/m·K) [11,36].

There are already studies about the thermal performance of renders with aerogel incorporation but the authors did not specify their mechanical strength [11,17]. In order to optimize the composition of mortars with aerogels, it is necessary to make changes in terms of binders, aggregates, water-binder ratios, and additions/admixtures and understand the influence of each component on the performance of these renders.

Aerogels continue to have high production costs, which are decisive in the use of these materials [37]. More productive ways of making aerogels have been studied; in particular faster and cheaper methods of drying [36] in order to minimize this problem. Once the high costs associated with their production are overcome, these materials will be an asset in finding competitive solutions of high thermal performance renders.

## 3. Experimental Work

In this experimental work 85 mortar mixes were prepared in the laboratory in several stages, changing the content of the binders matrix (cement; cement with aerial lime; cement with fly ash; cement with aerial lime, and fly ash), aggregates (silica sand, expanded clay, silica-based aerogel, expanded cork granules, and expanded perlite), and additions/admixtures (air entrainers, resins, and rheological agent). Due to the hydrophobicity of the aerogels, the water/binder ratio also changed in order to obtain homogeneous and workable mixes. Three cement types (CEM II/B-L 32,5N; CEM I 42,5R, and CEM I 52,5R) and four hybrid aerogel types (three commercial obtained through supercritical drying and one produced in the laboratory through subcritical drying—at atmospheric pressure [36]) were used. The three commercial aerogels were designated CA1, CA2, and CA3 and have the following bulk densities, respectively: 72.75, 62.78, and 66.96 kg/m^3^. The aerogel from subcritical drying was called HYB-C and its bulk density is 305.58 kg/m^3^. In order to optimize the aerogel content needed to determine the bulk density the procedure proposed in NP EN 1097-3 [38] was used. The bulk density test was performed using a small-scale recipient (0.027 L), with an interior diameter/interior depth ratio of 0.75, within the 0.5–0.8 range imposed by NP EN 1097-3 [38]. The maximum size of all aggregates was 2 mm, except for aerogel HYB-C, which was 0.5 mm and expanded perlite that was below 0.3 mm. The incorporation of aerogel has a significant influence on the thermal and mechanical properties of the mortars, contributing to a better thermal conductivity coefficient (lower values) but reducing the mechanical properties [37].

The objective of this study was to analyze mixes in order to obtain thermal mortars compromising the mechanical properties as little as possible. Table 1 generically shows the composition of the 85 mixes analyzed through the contents by mass of their components, which are detailed in Appendix (Table A1). The binder characterization according to the product technical sheet is presented in Table 2, and the particle size distribution of the silica sand, expanded clay, and expanded cork granules are presented in Table 3.

In order to respect thermal Classes T1 and T2, according to standard EN 998-1 [14], with thermal conductivity coefficients below 0.1 and 0.2 W/m·K, respectively, high contents of aerogel were incorporated. All of the renders selected contain 5% to 38% (in mass) of aerogel. However, aerogel’s contribution to thermal behavior is negatively affected by the other renders’ components density, such as silica sand aggregate or cement binder. To achieve this goal, renders must have a low density, so it was necessary to replace silica sand with lightweight aggregate, such as expanded clay, expanded cork granules, or expanded perlite, and replace part of the cement by aerial lime and/or fly ash. The incorporation of these materials in replacement of silica sand contributes to improving the thermal performance [19,24] but affects the renders’ mechanical strength [41].

Simultaneously, the content of air entrainers (olefin sulfonate, Na-salt), resins (styrene-acrylic copolymer), and a rheological agent (methyl hydroxyethyl cellulose) was also changed to improve the mixes’ properties. The incorporation of air entrainers intended to introduce pores in the mixes and, thus, benefit their thermal behavior. The powder or liquid resins were used to improve the bond between the various components. According to [42,43] resins act as a plasticizer, thus reducing the required mixing water content and improving the mixes’ internal bond. The rheological agent works as a water retainer conferring greater viscosity to the mix, which also allows greater homogeneity, better workability and a decrease in materials segregation [27], which are important when working with materials of different densities.

### 3.1. Render Production

**Figure 1:**
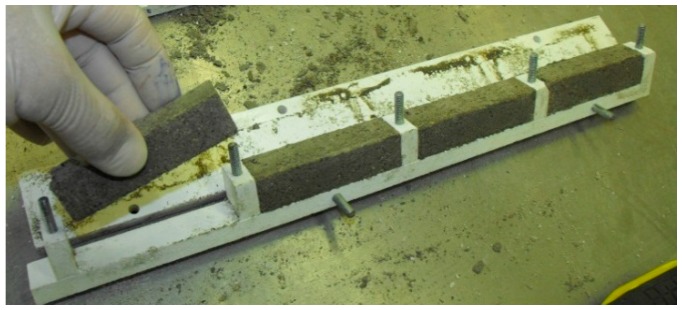
Small-scale prismatic moulds and specimens.

**Figure 2:**
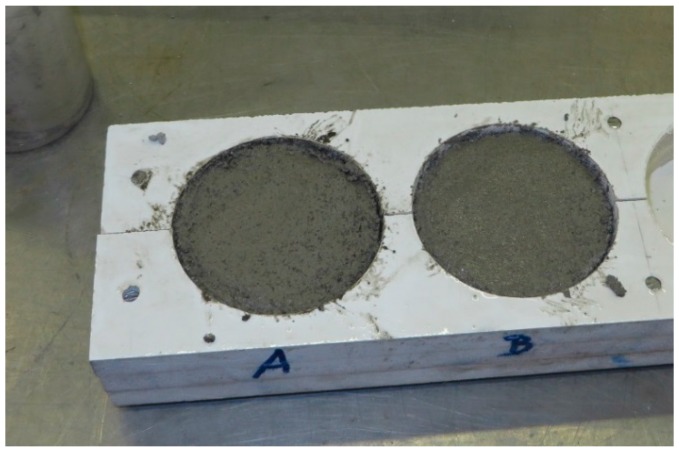
Small-scale cylindrical molds and specimens.

With the objective of reducing the aerogel consumption to prepare the laboratory specimens, due to its high cost and limitation to the production of large quantities in the laboratory, small-scale specimens were produced (Figure 1 and Figure 2), based on previous work [44].

In order to use the small-scale specimens, an analysis of the effects of changing the size and adapting the mixing and compacting procedures was made [44]. The results were acceptable in what concerns the correlation between the values from the various tests in standard specimens and small-scale specimens (coefficients of determination, *R*^2^, higher than 0.77) [44].

Due to the use of small-scale specimens, it was necessary to adapt the procedures, namely the mixing (made with an adapted drill) and compacting (made with a Ø4 mm metal rod) methods.

The mortars’ mixing was accomplished according to the following procedure:

- Pour the air entrainers into a mixing glass;
- Insert the aggregates in the same recipient;
- Pour 80% of the mixing water (when liquid resin was used; otherwise, pour 100% of the water);
- Stir the recipient in the vortex mixer device (model “VWR VV3”) at a moderate speed for 4 min;
- Insert the cement;
- Mix with the drill (at the minimum rotating speed possible) for 1 min;
- Scrape the left-over material and mix manually with a trowel;
- Add the resin previously mixed with 20% of the mixing water (when liquid resin was used);
- Add the rheological agent;
- Mix again with the drill (at the minimum rotating speed possible) for 1 min.

The specimens were molded and compacted manually, based on EN 1015-11 [45]. The curing and stocking of the specimens followed the same standard, *i.e.*, they were positioned inside a conditioned chamber at 20 ± 2 °C and 65% ± 5% relative humidity until testing. In the first seven days the specimens, still inside the molds, were wrapped in polyethylene bags. At 28 days, in the hardened state, the tests needed to determine the thermal conductivity and the compressive strength were performed.

### 3.2. Thermal Conductivity Test

In the thermal conductivity test, cylindrical specimens (Figure 2) were used, 20 mm high and with a 60 mm diameter. The testing conditions are defined in ISO 10456 [46]—23 °C and 50% relative humidity. The equipment used was ISOMET 2114 [47] which consists of a surface probe that analyses the response of the material to thermal impulses [48]. The heat flux impulses are realized through heating an electrical resistance inside the probe that is in contact with the specimen. Periodic registers are made as a function of time and the specimen’s temperature [19].

### 3.3. Compressive Strength

In order to evaluate the compressive strength indirectly through the dynamic modulus of elasticity [49], a non-destructive method was used. Based on the relationship obtained by Silva *et al.* [44], tests were performed in prismatic 20 × 20 × 80 mm^3^ specimens.

The dynamic modulus of elasticity was determined using the GrindoSonic MK5 “Industrial” equipment (J.W.LEMMENS N.V., Leuven, Belgium) and according to ASTM E 1876 [50].

## 4. Statistical Modelling

### 4.1. Principles

Multiple linear regression is one of the most used statistical tools in order to understand the influence of a set of (independent) variables on a given phenomenon or parameter, called the dependent variable [51,52,53]. This statistical technique intends to obtain the best model to describe the relationship between the dependent and the independent variables, as given by Equation (1):

(1) $$y=B_{0}+B_{1}\times x_{1}+B_{2}\times x_{2}+\cdots +B_{k}\times x_{k}+\varepsilon$$

where y represents the dependent variable, here the thermal conductivity or the compressive strength; B_0_, B_1_, B_2_, ..., B_k_ are the coefficients; x_1_, x_2_, ... x_k_ are the independent variables; and ε the errors associated with the model.

Multiple linear regression can be applied in two ways, one in which the dependent variable can be determined through various independent variables (prediction), and another through the reverse process in which the influence of each of the independent variables on explaining the dependent variable is determined [51,54,55].

In this study, multiple linear regressions are used to statistically model the thermal and mechanical performance of aerogel-based renders, established respectively for the thermal conductivity and the compressive strength. They were both considered dependent variables and were analyzed separately. Since it is intended to understand the influence of the various components of the renders on their thermal and mechanical performance, the independent variables are these components’ content: cement, fly ash, aerial lime, silica sand, expanded clay, silica-based aerogel, expanded cork granules, expanded perlite, air entrainers, rheological agent, and resin. The water/binder ratio was also analyzed but it was not considered statistically significant.

The statistical analysis is made using the software SPSS (Statistical Package for Social Sciences). In the model’s definition a stepwise technique [51,56] is applied, ensuring that all the assumptions related to the statistical significance of the model are fulfilled, eliminating multicollinearity effects (*i.e.*, removing pseudo-independent or intercorrelated variables that can jeopardize the results of the Multiple Linear Regression analysis performed) [57]. This stepwise technique allows identifying all the statistically-relevant variables for the description of the phenomena under analysis. In the multiple linear regression equation that characterizes the model, the independent variables sequence follows a decreasing importance trend in terms of the relevance to the dependent variable analyzed [52].

The following coefficients are relevant in terms of the analysis of the quality of the model from a statistical point of view:

- *r* (Pearson correlation coefficient)—measures the correlation degree between variables and ranges from −1 and 1; a correlation coefficient equal to 1 in absolute value indicates a “perfect” linear correlation between the variables (*i.e.*, revealing that all the points in the dataset coincide with the regression line);
- *R*^2^ (determination coefficient)—measures the fraction of variability of the dependent variable that may be explained by the obtained regression model;
- Adjusted *R*^2^ (adjusted determination coefficient)—the determination coefficient increases as new independent variables are added; thus, the adjusted determination coefficient only increases if the addition of a new variable to the regression model leads to an improvement of its adjustment;
- Standard-error—represents the standard deviation of the error.

### 4.2. Selected Sample

This study intended to develop statistical models of the thermal and mechanical performance (through the thermal conductivity coefficient and compressive strength, respectively) of aerogel-based renders, built from a sample of 85 cases (composition on Table 1).

The sample is quite heterogeneous, as a result of the main objective of the research project, which was to optimize the content of aerogels on aerogel-based renders and reach a compromise between thermal conductivity and mechanical properties.

Four models were produced for thermal conductivity: in the first one (Model 1) all case studies containing a value of the parameter under analysis were used (N = 85 cases) and the other three (Models 2–4) included each one of a single type of aerogel: CA2 (N = 16 cases), CA3 (N = 41 cases), and HYB-C (N = 28 cases). Only one model (Model 5) was produced for compressive strength (N = 46 cases) since there were not enough data to establish the other models.

### 4.3. Resulting Models

#### 4.3.1. Model 1 (Whole Sample)

Model 1, described by Equation (2), intends to estimate the thermal conductivity of aerogel-based mortars, using the whole sample.

(2) λ=0.163−0.002L+0.003SS−0.005CA2−0.002CV−0.003CA3−0.021P−0.035AE

where L represents the aerial lime content; SS the silica sand content; CA2 the CA2 aerogel content; CV the fly ash content; CA3 the CA3 aerogel content; P the expanded perlite content; and AE the air entrainers content.

Table 4 summarizes the model, whose determination coefficient (*R*^2^) is 0.738, *i.e.*, 73.8% of the variability associated to the thermal conductivity coefficient is explained by the seven independent variables present in the equation and the remaining 26.2% are due to other non-analyzed causes.

**Figure 3:**
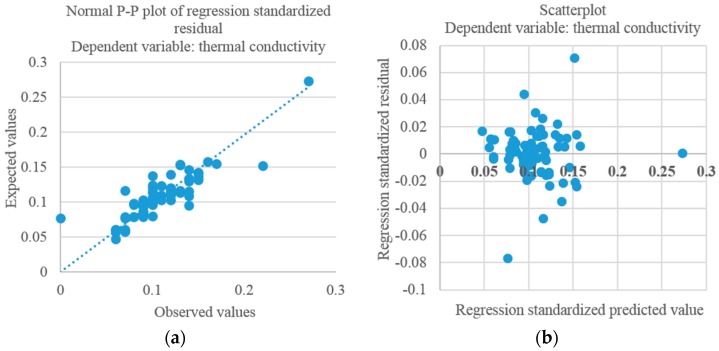
Normal probability plot of residuals for Model 1 (thermal conductivity). (**a**) Normal P-P plot of regression standardized residual; (**b**) Scatterplot.

The coefficients are all negative except for the silica sand content (0.003), indicating that the presence of silica sand leads to an increase of the values of the thermal conductivity. A *p*-value of 3.24 × 10^−20^ is achieved for Model 1 and Figure 3 shows the residual plots for this model.

With these results, it is possible to establish a general Equation (3), for aerogel that is incorporated in each render:

(3) λ=0.163−0.002L+0.003SS−0.002FA−0.021P−0.035AE−Aerogel

where L represents the aerial lime content; SS the silica sand content; FA the fly ash content; P the expanded perlite content; AE the air entrainers content; and Aerogel the aerogel content. The variable Aerogel can be replaced with the relationship presented in Equation (4):

(4) Aerogel=0.005CA2+0.003CA3

#### 4.3.2. Thermal Conductivity of Aerogel-Based Mortars in Function of Aerogel Type

After defining an equation for the thermal conductivity using all renders, it was deemed necessary to establish models as a function of the type of aerogel used. The results for the renders with CA2 aerogel were inconclusive, because of the size of the sample (only 16 case studies) and its homogeneity, *i.e.*, using renders with very similar compositions, thus preventing the identification of the variables with predictive power in terms of the thermal conductivity of renders.

#### 4.3.3. Model 2 (CA3 Aerogel)

The third model obtained analyzes the mortars with incorporation of the CA3 aerogel (N = 41 cases), leading to Equation (5):

(5) λ=0.143−0.002L−0.002FA−0.089AE

where L represents the aerial lime content; FA the fly ash content; and AE the air entrainers content. In this model the *R*^2^ obtained was 0.456, *i.e.*, only 45.6% of the variability of the thermal conductivity can be explained by the variables considered. A *p*-value of 4.42 × 10^−5^ is achieved for this model. Therefore, this model is only indicative, since this section intends to provide some indications related with the thermal conductivity of aerogel-based mortars as a function of aerogel type. For this type of aerogel, the results should be carefully analyzed.

#### 4.3.4. Model 3 (HYB-C Aerogel)

As for the hybrid aerogel subcritical (HYB-C), 28 case studies were analyzed, leading to Equation (6):

(6) λ=0.177+0.003SS−0.010FA−0.055AE

where SS represents the silica sand content; FA the fly ash content; and AE the air entrainers content. In this regression model the *R*^2^ obtained was 0.752, showing that it is adequate. 75.2% of the variability associated to the thermal conductivity coefficient is explained by the variables considered, and the remaining 24.8% are due to other causes not included in this analysis. Model 4 establishes negative coefficients, *i.e.*, favorable to reduce the thermal conductivity, for fly ash and air entrainers and, as for Model 1, silica sand has a positive coefficient (unfavorable effect). For this model, a *p*-value of 1.87 × 10^−7^ is obtained.

#### 4.3.5. Model 4 (Compressive Strength)

The analysis of the compressive strength yielded one model only since there were not enough data to perform further analyses. Forty-six case studies were relevant to the model and led to Equation (7). The summary of the model is presented in Table 5, revealing a determination coefficient of 0.851, *i.e.*, a good correlation between the sample analyzed and the model proposed.

(7) $$f_{c}=0.966-0.049\text{HYBC}+0.056\text{EC}$$

where HYBC represents the HYB-C aerogel content; and EC the expanded clay content. The positive coefficient of the expanded clay represents the positive effect of its incorporation for the compressive strength.

**Figure 4:**
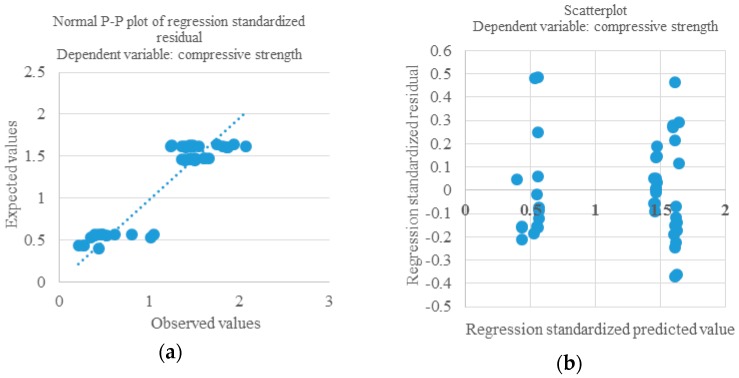
Normal probability plot of residuals for Model 4 (compressive strength). (**a**) Normal P-P plot of regression standardized residual; (**b**) Scatterplot.

**Figure 5:**
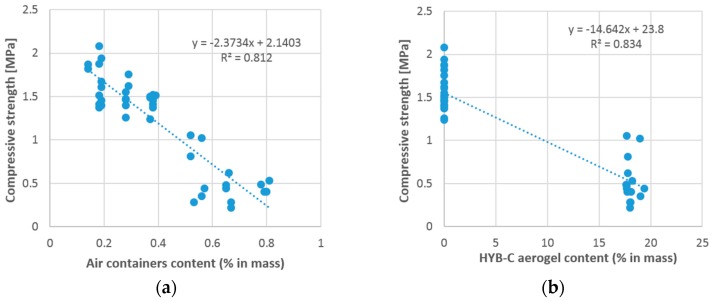
Compressive strength in function of the: (**a**) the air entrainers content (% in mass); and (**b**) HYB-C aerogel content (% in mass).

It was found that the incorporation of both aerogel HYB-C and air entrainers leads to a decrease of the thermal renders’ strength. However, in the sample analyzed, the HYB-C aerogel content compromises the air entrainers’ content that needs to be added to the composition of the thermal renders. Therefore, these two variables explain/are related with each other, *i.e.*, they cannot both function as independent variables to explain the variability of compressive strength. For example, the higher amount of air entrainers corresponds to this type of aerogel, while lower amounts correspond to other types of aerogel. This model leads to a *p*-value of 2.91 × 10^−17^ and Figure 4 presents the normal probability plot of residuals for this model. Figure 5 shows the relationship between the renders´ compressive strength and their content (% in mass) of air entrainers and HYB-C aerogel, respectively.

## 5. Results Discussion

The use of linear regression analyses allows determining more objectively the importance and influence of any factor on a given phenomenon. The results depend essentially on the quality of the sample [49], namely its heterogeneity, *i.e.*, usually, more and better data lead to more reliable results. This research work is based on laboratory data, obtained through a long experimental campaign, analyzing an innovative material with the addition of other components that allow obtaining aerogel-based renders’ with good thermal and mechanical performance.

### 5.1. Influence of the Components on the Thermal Conductivity Coefficient (λ)

From the equation obtained in Section 4.3.1, it is found that the independent variables that influence the thermal conductivity are: aerial lime, silica sand, fly ash, expanded perlite, air entrainers, and aerogel content (Equation (3)).

Aerial lime is known to increase renders’ porosity, leading to lower render density and, consequently, lower thermal conductivity [19,24]. As expected, in this analysis aerial lime confirms that trend with a coefficient of −0.002 in Equation (3).

The positive coefficient of silica sand was also expected, since this material has a high density that does not favor a decrease of the thermal conductivity. Therefore, in thermal renders sand is often replaced with lightweight aggregates. As for aerogel, and as expected, it is found to be one of the variables that contribute to a better thermal performance of the renders [1,11,28]. Fly ash influences positively (*i.e.*, reduces) the thermal conductivity [10], and the −0.002 coefficient in Equation (3) confirms that characteristic. It is also found that expanded perlite (with a coefficient of −0.021 in Equation (3)), a material with low density, decreases the thermal conductivity coefficient. Since air entrainers are important to create pores within the mortar, its positive influence on thermal conductivity was expected. Unexpectedly, the expanded cork granules were not relevant in this analysis. It was expected that this material with great insulation potential would come up in the equation with a negative coefficient. The explanation for this lack of a clear trend may be the fact that, when expanded cork granules are used, expanded clay is also used which, despite being a lightweight aggregate, induces an increase of density when compared with expanded cork granules or aerogel.

Some other situations raise this issue, such as the case of resins and the rheological agent, whose influence was not relevant in this model, even though other studies concluded that the thermal conductivity decreases considerably when they are incorporated in mortars [26,27].

In Section 4.3.3 (concerning the CA3 aerogel), negative coefficients were obtained for aerial lime, fly ash, and air entrainers, according to a decreasing ranking of their influence as independent variables in Equation (5). In the model described in Section 4.3.4 (concerning the HYB-C aerogel) silica sand has a positive coefficient, and fly ash and air entrainers have negative coefficients, according to a decreasing ranking of their influence as independent variables in Equation (6).

In the three equations obtained for thermal conductivity the conclusions were coherent: aerial lime, fly ash, and air entrainers influence positive thermal conductivity. According to various authors [10,25], fly ash and lime can be used as a partial replacement of cement in order to reduce the thermal conductivity of mortars. The incorporation of these materials allows obtaining lighter renders, with higher porosity, thus decreasing the thermal conductivity coefficient [19,25,26].

### 5.2. Influence of the Components on Compressive Strength (Cs)

From the model devised in Section 4.3.5 for compressive strength the following independent variables influencing that parameter were obtained: aerogel and expanded clay (Equation (7)). The cement type necessarily has a significant influence on this mechanical performance characteristic of the renders but was not identified as an explaining variable because around 97% of the 46 cases analyzed contained CEM I 42,5R, *i.e.*, the sample was homogeneous in terms of this binder.

**Figure 6:**
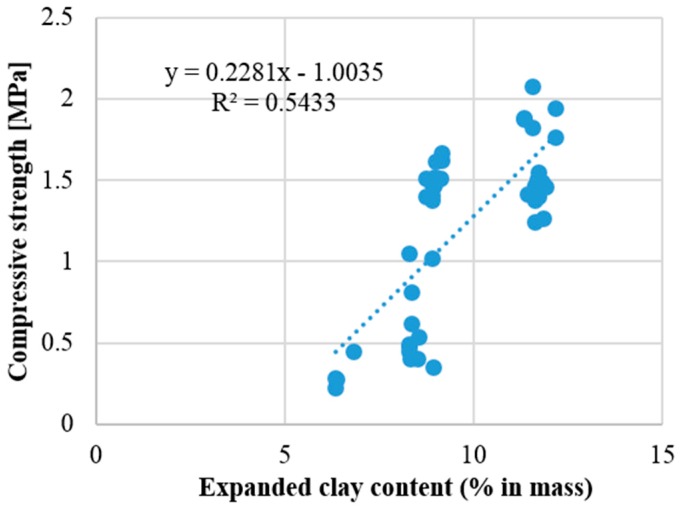
Compressive strength in function of the expanded clay content (% in mass).

Concerning the model related to the compressive strength of the analyzed renders, the HYB-C aerogel content is the most relevant variable with a determination coefficient of 0.834 (Figure 5b). The other variable included in this model is the expanded clay content; the simple linear regression relationship between compressive strength and expanded clay are presented in Figure 6. This analysis, regardless of its statistical significance, reveals that increasing the expanded clay content increase the renders’ strength [19,22].

Expanded clay is a lightweight aggregate with higher density when compared with aerogel or cork aggregates [21,22], also the relatively high structural strength of expanded clay aggregates can justify the performance in terms of compressive strength [21]. As expected the contribution of the aerogel and air entrainers is unfavorable to the compressive strength (Figure 5).

### 5.3. Relative Importance of the Components

In addition to the influence of the components on the thermal and mechanical performance of the aerogel-based mortars, this study intends to understand their relative importance for each of these properties.

**Figure 7:**
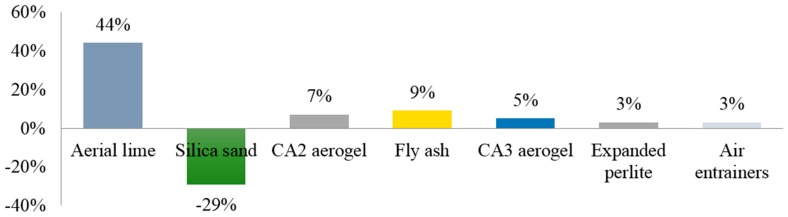
Relative importance of the components on the thermal conductivity of aerogel-based renders.

Figure 7 allows understanding the relative importance of the components on the thermal conductivity. Aerial lime assumes the main role (44%) followed by silica sand (29%). It is noted that, unlike aerial lime, silica sand’s role is unfavorable and, therefore, its incorporation in thermal renders must be dealt with carefully, since its effects are significant and unwanted. It increases the renders’ bulk density contrarily to lightweight aggregates, such as cork or expanded clay.

The other components with positive influence on the thermal conductivity are CA2 aerogel (with a bulk density of 62.78 kg/m^3^), fly ash, CA3 aerogel (bulk density of 66.96 kg/m^3^), expanded perlite, and air entrainers.

The importance of fly ash was expected to be greater than that of aerial lime, but in this set of renders this pozzolanic component was only used together with cement, which offset its expected effects.

Concerning the compressive strength of aerogel-based renders, the content in mass of HYB-C aerogel has the greatest relative importance (98%) and expanded clay contributed with 2% only to the predictive capacity of the model. This model also allows understanding that the aerogel and air entrainers are unfavorable to the compressive strength. The cases analyzed with HYB-C aerogel have large contents of air entrainers, which may have led to their negative influence on compressive strength in the model. The content of this component in renders must be the adequate one to guarantee a good thermal performance without compromising the mechanical behavior.

The simultaneous presence of various components makes this analysis very complex. It is clear that the results are always a mix of various actions and that the best solution lies in a balance of the contents.

## 6. Conclusions

The statistical modelling of the 85 aerogel-based renders’ results obtained in the laboratory allowed both identifying the components with more influence on the thermal and mechanical performance and drawing conclusions on the type of combinations of materials needed to optimize these thermal renders (with thermal conductivity below 0.1 W/m·K).

According to the first model, the variables influential on the thermal conductivity coefficient are: aerial lime, silica sand, aerogel, fly ash, expanded perlite, and air entrainers. As expected, only silica sand is the component that affects negatively (*i.e.*, increases) this coefficient. Contrary to expectations, some components were not relevant to the analysis of the thermal conductivity, e.g., the expanded cork granules. Future studies with fewer variables are considered important to prevent interference resulting from too many components acting simultaneously. The conclusions drawn from the three proposed models for the thermal conductivity coefficient are coherent.

According to the model proposed for compressive strength, the independent variables with influence on this parameter were aerogel and expanded clay. Expanded clay leads to an increase in the renders’ compressive strength; in other hand, aerogel has a negative effect to the development of compressive strength. Furthermore, the air entrainers, whose content depends on the type of aerogel used (commercial or produced in laboratory), contribute to a reduction of the strength of the analyzed renders.

Based on these results concerning the relative importance of the various components on both properties, it is concluded that the optimal solutions lie in adequate proportioning in order to reach a balance between these normally-conflicting properties of renders. Aerial lime assumes the main role on thermal conductivity, followed by silica sand, CA2 supercritical aerogel, fly ash, CA3 supercritical aerogel, expanded perlite, and air entrainers. According to the relative importance of the components on compressive strength, aerogel comes out on top, followed by expanded clay (with residual relevance).

This work provides useful information for aerogel-based thermal render formulation and optimization, guaranteeing adequate mechanical characteristics (values above 0.4 N/mm^2^, according to EN998-1 [14]), reducing costs production (using subcritical aerogel and incorporating other lightweight aggregates), and reducing environmental impacts (partial replacement of cement by aerial lime and/or fly ash).

## Figures and Tables

**Table 1:** Composition of the 85 mortars produced (component percentages by mass).

Components	Average Value (%)	Range (%)
Cement CEM II/B-L 32,5N	40.45	8.46–53.11
Cement CEM I 42,5R	16.48	8.12–19.20
Cement CEM I 52,5R	8.12	-
Fly ash	3.80	2.61–23.77
Aerial Lime	15.81	10.42–41.16
Water	40.82	21.43–53.03
Silica sand (<2 mm)	42.20	-
Expanded clay (0.5–2 mm)	10.42	6.37–25.22
Aerogel HYB-C subcritical (<0.5 mm)	24.02	14.49–38.34
Aerogel CA1 supercritical (<2 mm)	13.71	-
Aerogel CA2 supercritical (<2 mm)	9.36	5.08–12.54
Aerogel CA3 supercritical (<2 mm)	7.19	5.29–12.76
Expanded cork granules (0.5–2 mm)	1.59	1.00–4.08
Expanded perlite (1–300 µm)	0.71	0.54–0.78
Air entrainers (olefin sulphonate)	0.36	0.01–0.85
Powder resin	0.81	0.52–1.45
Liquid resin (copolymer styrene acrylic)	3.69	1.51–8.37
Rheological agent (methyl hydroxyethyl cellulose)	0.03	0.02–0.05
Water/binder ratio	1.14	0.61–1.75

**Table 2:** Composition and chemical characterization of the cement.

Type of Cement	Composition	Properties/Testing Method/Specifications According to Standard
CEM I 42,5R	95%–100% of clinker 0%–5% of minor component	Sulfate content (SO_3_)/EN 196-2 [39]/≤4.0% Chloride content (Cl)/EN 196-21 [40]/≤0.1%
CEM I 52,5R	95%–100% of clinker 0%–5% of minor component	Sulfate content (SO_3_)/EN 196-2 [39]/≤4.0% Chloride content (Cl)/EN 196-21 [40]/≤0.1%
CEM II/B-L 32,5N	65%–79% of clinker 21%–35% of limestone 0%–5% of minor component	Sulfate content (SO_3_)/EN 196-2 [39]/≤3.5% Chloride content (Cl)/EN 196-21 [40]/≤0.1%
Fly ash	SiO_2_; Al_2_O_3_; Fe_2_O_3_; CaO	-
Aerial Lime	Calcium hydroxide content (Ca(OH)_2_) ≥93% Magnesium content (MgO) ≤3%	-

**Table 3:** Particle size distribution of silica sand, expanded clay, and expanded cork granules.

Material	Size (mm)					
	<0.063	0.063 to 0.125	0.125 to 0.250	0.250 to 0.500	0.500 to 1.000	1.000 to 2.000
Silica sand	0.5	0.5	17.0	59.0	22.0	1.0
Expanded clay	-	-	-	-	17.7	82.3
Expanded cork granules	-	-	-	-	17.7	82.3

**Table 4:** Summary of Model 1 to estimate the thermal conductivity of aerogel-based renders.

Step ^a^	*r*	*R*^2^	Adjusted *R*^2^	Square Root of the Average Square Error
1	0.567 ^b^	0.322	0.314	0.029
2	0.732 ^c^	0.536	0.525	0.024
3	0.869 ^d^	0.592	0.577	0.023
4	0.810 ^e^	0.657	0.640	0.021
5	0.835 ^f^	0.697	0.678	0.020
6	0.847 ^g^	0.718	0.696	0.019
7	0.859 ^h^	0.738	0.714	0.019

^a^ Dependent variable: Thermal conductivity coefficient; ^b^ Independent variables: (constant), aerial lime; ^c^ Independent variables: (constant), aerial lime, silica sand; ^d^ Independent variables: (constant), aerial lime, silica sand, CA2; ^e^ Independent variables: (constant), aerial lime, silica sand, CA2, fly ash; ^f^ Independent variables: (constant), aerial lime, silica sand, CA2, fly ash, CA3; ^g^ Independent variables: (constant), aerial lime, silica sand, CA2, fly ash, CA3, expanded perlite; ^h^ Independent variables: (constant), aerial lime, silica sand, CA2, fly ash, CA3, expanded perlite, air entrainers.

**Table 5:** Summary of Model 4 to estimate the compressive strength of aerogel-based mortars.

Step ^a^	*r*	*R*^2^	Adjusted *R*^2^	Square Root of the Average Square Error
1	0.914 ^b^	0.835	0.831	0.224
2	0.922 ^c^	0.851	0.844	0.216

^a^ Dependent variable: Compressive strength; ^b^ Independent variables: (constant), HYB-C; ^c^ Independent variables: (constant), HYB-C, expanded clay.

## Appendix A

**Table A1:** Mortar compositions (%wt.); water/binder ratio; tests at 28 days.

MD	CEM II/B-L 32,5N	CEM I 42,5R	CEM I 52,5R	FA	L	W/B	SS	EC	HYB-C	CA1	CA2	CA3	ECG	P	AE	PR	LR	RA	W	λ	Cs
1	47.54	0	0	0	0	0.85	0	0	0	0	0	11.74	0	0	0.24	0	0	0.03	40.41	0.068	-
2	22.2	0	0	0	22.2	1	0	0	0	0	0	10.96	0	0	0.22	0	0	0.03	44.39	0.073	-
3	23.77	0	0	23.77	0	0.85	0	0	0	0	0	11.74	0	0	0.24	0	0	0.03	40.41	0.071	-
4	21.26	0	0	4.23	17.03	1.1	0	0	0	0	0	10.5	0	0	0.21	0	0	0.03	46.77	0.069	-
5	0	0	0	0	39.98	1.25	0	0	0	0	0	9.87	0	0	0.2	0	0	0.03	49.97	0.064	-
6	8.46	0	0	0	34.05	1.1	0	0	0	0	0	10.5	0	0	0.21	0	0	0.03	46.77	0.061	0.45
7	0	8.12	0	0	32.67	1.3	0	0	0	0	0	10.07	0	0	0.2	0	0	0.03	53.03	0.059	0.57
8	0	0	8.12	0	32.67	1.3	0	0	0	0	0	10.07	0	0	0.2	0	0	0.03	53.03	0.057	0.53
9	0	0	0	0	41.16	1.2	0	0	0	0	0	6.1	3.15	0	0.2	0	0	0.03	49.4	0.069	-
10	48.77	0	0	0	0	0.8	0	0	0	0	0	12.07	0	0	0.25	0	0	0	39.01	0.129	-
11	49.87	0	0	0	0	0.75	0	0	0	0	0	12.34	0	0	0.25	0	0	0	37.41	0.131	-
12	51.57	0	0	0	0	0.66	0	0	0	0	0	12.76	0	0	0.26	0	2.62	0.04	34.03	0.125	-
13	48.18	0	0	0	0	0.8	0	0	0	0	0	11.92	0	0	0.24	0	2.41	0.02	38.54	0.142	-
14	52.81	0	0	0	0	0.61	0	0	0	0	12.54	0	0	0	0.27	0	5.28	0.04	32.21	0.092	-
15	51.2	0	0	0	0	0.66	0	0	0	0	12.15	0	0	0	0.26	0	5.12	0.04	33.79	0.091	-
16	51.98	0	0	0	0	0.66	0	0	0	0	12.05	0	0	0	0.26	0	2.64	0.04	34.31	0.140	-
17	53.11	0	0	0	0	0.66	0	0	0	0	7.4	0	4.08	0	0.27	0	0	0	35.05	0.117	-
18	50.54	0	0	0	0	0.76	0	0	0	0	7.04	0	3.88	0	0.26	0	0	0	38.41	0.104	-
19	47.37	0	0	0	0	0.86	0	0	0	0	6.6	0	3.64	0	0.24	0	2.88	0.04	40.74	0.109	-
20	46.48	0	0	0	0	0.86	0	0	0	0	6.48	0	3.57	0	0.23	0	4.71	0.04	39.97	0.108	-
21	46.05	0	0	0	0	0.86	0	0	0	0	6.42	0	3.54	0	0.23	0	8.37	0.03	39.6	0.100	-
22	50.45	0	0	0	0	0.81	0	0	0	0	7.03	0	0	0	0.25	0	2.52	0.04	40.87	0.115	-
23	39.32	0	0	0	0	0.76	0	25.22	0	0	5.48	0	0	0	0.2	0	0	0	29.88	0.144	-
24	36.41	0	0	0	0	0.86	0	23.36	0	0	5.08	0	0	0	0.18	0	3.68	0.03	31.32	0.154	-
25	52.94	0	0	0	0	0.66	0	0	0	0	12.3	0	0	0	0	0	0	0	34.94	0.100	-
26	52.93	0	0	0	0	0.66	0	0	0	0	12.3	0	0	0	0.01	0	0	0	34.94	0.111	-
27	52.93	0	0	0	0	0.66	0	0	0	0	12.3	0	0	0	0.03	0	0	0	34.93	0.104	-
28	52.91	0	0	0	0	0.66	0	0	0	0	12.29	0	0	0	0.05	0	0	0	34.92	0.093	-
29	52.8	0	0	0	0	0.66	0	0	0	0	12.27	0	0	0	0.27	0	0	0	34.85	0.086	-
30	36.72	0	0	0	0	0.95	0	0	24.84	0	0	0	2.81	0	0.73	0	0	0.03	34.89	0.103	-
31	29.56	0	0	0	0	1.05	0	18.81	19.99	0	0	0	0	0	0.59	0	0	0.02	31.04	0.154	-
32	21.43	0	0	0	0	1	42.2	0	14.49	0	0	0	0	0	0.43	0	0	0.02	21.43	0.273	-
33	32.93	0	0	0	0	0.9	0	0	37.22	0	0	0	0	0	0.25	0	0	0	29.64	0.169	-
34	30.85	0	0	0	0	1.1	0	0	34.87	0	0	0	0	0	0.31	0	0	0.02	33.93	0.223	-
35	29.94	0	0	0	0	1.2	0	0	33.84	0	0	0	0	0	0.3	0	0	0	35.92	0.132	-
36	31.79	0	0	0	0	1	0	0	35.93	0	0	0	0	0	0.48	0	0	0	31.79	0.137	-
37	31.74	0	0	0	0	1	0	0	35.88	0	0	0	0	0	0.64	0	0	0	31.74	0.146	-
38	33.92	0	0	0	0	0.8	0	0	38.34	0	0	0	0	0	0.69	0	0	0	27.13	0.118	-
39	31.69	0	0	0	0	1	0	0	35.82	0	0	0	0	0	0.8	0	0	0	31.69	0.147	-
40	33.86	0	0	0	0	0.8	0	0	38.27	0	0	0	0	0	0.85	0	0	0	27.09	0.139	-
41	29.76	0	0	0	0	1.2	0	0	33.6	0	0	0	0	0	0.15	0	1.51	0.02	35.71	0.164	-
42	0	14.07	0	2.82	11.25	1.5	0	8.96	19.04	0	0	0	1.08	0	0.56	0	0	0.02	42.2	0.121	0.35
43	0	14.31	0	2.86	11.44	1.5	0	6.83	19.37	0	0	0	1.1	0.58	0.57	0	0	0.02	42.93	0.120	0.44
44	0	13.99	0	2.8	11.19	1.5	0	8.91	18.94	0	0	0	1.07	0	0.56	0.56	0	0.02	41.97	0.130	1.02
45	0	13.14	0	2.63	10.51	1.75	0	8.37	17.79	0	0	0	1.01	0	0.52	0	0	0.04	45.98	0.115	0.81
46	0	13.34	0	2.67	10.67	1.75	0	6.37	18.07	0	0	0	1.02	0.54	0.53	0	0	0.04	46.7	0.104	0.28
47	0	13.07	0	2.62	10.45	1.75	0	8.32	17.69	0	0	0	1	0	0.52	0.52	0	0.04	45.74	0.121	1.05
48	0	13.12	0	2.63	10.5	1.75	0	8.36	17.77	0	0	0	1.01	0	0.66	0	0	0.02	45.93	0.101	0.62
49	0	13.33	0	2.67	10.66	1.75	0	6.37	18.04	0	0	0	1.02	0.54	0.67	0	0	0.02	46.65	0.101	0.28
50	0	13.06	0	2.61	10.45	1.75	0	8.32	17.68	0	0	0	1	0	0.65	0.52	0	0.02	45.71	0.120	0.44
51	0	13.12	0	2.63	10.49	1.75	0	8.36	17.76	0	0	0	1.01	0	0.66	0	0	0.04	45.92	0.100	-
52	0	13.33	0	2.67	10.66	1.75	0	6.37	18.04	0	0	0	1.02	0.54	0.67	0	0	0.04	46.64	0.094	0.22
53	0	13.05	0	2.61	10.44	1.75	0	8.31	17.67	0	0	0	1	0	0.65	0.52	0	0.04	45.68	0.119	0.48
54	0	13.46	0	2.69	10.77	1.65	0	8.57	18.22	0	0	0	1.03	0	0.81	0	0	0.02	44.42	0.139	0.53
55	0	13.39	0	2.68	10.71	1.65	0	8.52	18.12	0	0	0	1.03	0	0.8	0.53	0	0.02	44.18	0.112	0.40
56	0	13.1	0	2.62	10.48	1.75	0	8.34	17.74	0	0	0	1	0	0.79	0	0	0.04	45.86	0.123	0.40
57	0	13.03	0	2.61	10.42	1.75	0	8.3	17.64	0	0	0	1	0	0.78	0.52	0	0.04	45.62	0.107	0.49
58	51.01	0	0	0	0	0.66	0	0	0	13.71	0	0	0	0	0.26	0	2.59	0.04	33.66	0.130	-
59	0	19.12	0	3.83	15.3	1.1	0	12.18	0	0	0	5.67	1.47	0	0.19	0	0	0.03	42.07	0.104	1.94
60	0	18.42	0	3.69	14.73	1.2	0	11.73	0	0	0	5.46	1.41	0	0.19	0	0	0.05	44.21	0.102	1.40
61	0	19.12	0	3.83	15.29	1.1	0	12.17	0	0	0	5.67	1.47	0	0.29	0	0	0.03	42.06	0.103	1.76
62	0	18.4	0	3.68	14.72	1.2	0	11.72	0	0	0	5.46	1.41	0	0.28	0	0	0.05	44.17	0.101	1.55
63	0	18.74	0	3.75	14.99	1.15	0	11.93	0	0	0	5.56	1.44	0	0.38	0	0	0.03	43.1	0.09	1.46
64	0	18.39	0	3.68	14.71	1.2	0	11.71	0	0	0	5.45	1.41	0	0.37	0	0	0.05	44.13	0.089	1.50
65	0	18.29	0	3.66	14.63	1.2	0	11.65	0	0	0	5.43	1.4	0	0.18	0.73	0	0.03	43.91	0.086	1.37
66	0	17.96	0	3.59	14.36	1.25	0	11.44	0	0	0	5.33	1.38	0	0.18	0.72	0	0.05	44.9	0.096	1.41
67	0	18.62	0	3.73	14.89	1.15	0	11.85	0	0	0	5.52	1.43	0	0.28	0.74	0	0.03	42.82	0.079	1.26
68	0	18.27	0	3.66	14.61	1.2	0	11.63	0	0	0	5.42	1.4	0	0.28	0.73	0	0.05	43.85	0.09	1.47
69	0	18.6	0	3.72	14.88	1.15	0	11.84	0	0	0	5.52	1.43	0	0.37	0.74	0	0.03	42.78	0.086	1.49
70	0	18.25	0	3.65	14.6	1.2	0	11.62	0	0	0	5.41	1.4	0	0.37	0.73	0	0.05	43.81	0.082	1.24
71	0	19.2	0	3.84	15.36	1.15	0	9.17	0	0	0	5.7	1.47	0.78	0.19	0	0	0.03	44.16	0.093	1.67
72	0	18.83	0	3.77	15.06	1.2	0	9	0	0	0	5.59	1.44	0.77	0.19	0	0	0.05	45.2	0.089	1.61
73	0	19.18	0	3.84	15.34	1.15	0	9.16	0	0	0	5.69	1.47	0.78	0.29	0	0	0.03	44.12	0.097	1.62
74	0	18.81	0	3.77	15.05	1.2	0	8.99	0	0	0	5.58	1.44	0.77	0.28	0	0	0.05	45.15	0.089	1.47
75	0	18.8	0	3.76	15.03	1.2	0	8.98	0	0	0	5.58	1.44	0.76	0.38	0	0	0.05	45.11	0.095	1.52
76	0	18.7	0	3.74	14.95	1.2	0	8.93	0	0	0	5.55	1.43	0.76	0.19	0.75	0	0.03	44.87	0.094	1.46
77	0	18.35	0	3.67	14.67	1.25	0	8.76	0	0	0	5.44	1.41	0.75	0.18	0.73	0	0.05	45.86	0.088	1.51
78	0	18.68	0	3.74	14.94	1.25	0	8.92	0	0	0	5.54	1.43	0.76	0.28	0.75	0	0.03	46.7	0.087	1.47
79	0	18.33	0	3.67	14.66	1.25	0	8.76	0	0	0	5.44	1.41	0.75	0.28	0.73	0	0.05	45.82	0.091	1.40
80	0	18.66	0	3.73	14.93	1.2	0	8.91	0	0	0	5.54	1.43	0.76	0.38	0.75	0	0.03	44.79	0.079	1.37
81	0	18.66	0	3.73	14.92	1.2	0	8.91	0	0	0	5.53	1.43	0.76	0.38	0.75	0	0.05	44.77	0.082	1.41
82	0	18.16	0	3.63	14.52	1.2	0	11.56	0	0	0	5.39	1.39	0	0.18	1.45	0	0.03	43.58	0.103	2.08
83	0	17.83	0	3.57	14.26	1.25	0	11.35	0	0	0	5.29	1.37	0	0.18	1.42	0	0.05	44.57	0.100	1.88
84	0	18.17	0	3.64	14.53	1.2	0	11.57	0	0	0	5.39	1.39	0	0.14	1.45	0	0.03	43.6	0.100	1.82
85	0	17.83	0	3.57	14.26	1.25	0	11.36	0	0	0	5.29	1.37	0	0.14	1.43	0	0.05	44.58	0.104	1.87

Legend: MD—mortar designation; CEM II/B-L 32,5N—cement CEM II/B-L 32,5N; CEM I 42,5R—cement CEM I 42,5R; CEM I 52,5R—cement CEM I 52,5R; FA—fly ash; L—aerial lime; W/B—water/binder ratio; SS—silica sand; EC—expanded clay; HYB-C—aerogel HYB-C subcritical; CA1—aerogel CA1 supercritical; CA2—aerogel CA2 supercritical; CA3—aerogel CA3 supercritical; ECG—expanded cork granules; P—expanded perlite; AE—air entrainers; PR—powder resin; LR—liquid resin; RA—rheological agent; W—water; λ—thermal conductivity (W/m·K) at 28 days; Cs—compressive strength (MPa) at 28 days.
